# The Effect of Depressive Symptoms on the Association between Gluten-Free Diet Adherence and Symptoms in Celiac Disease: Analysis of a Patient Powered Research Network

**DOI:** 10.3390/nu10050538

**Published:** 2018-04-26

**Authors:** Andrew M. Joelson, Marilyn G. Geller, Haley M. Zylberberg, Peter H. R. Green, Benjamin Lebwohl

**Affiliations:** 1 Department of Internal Medicine, New York Presbyterian Hospital, New York, NY 10032, USA; amj9033@nyp.org; 2 Celiac Disease Foundation, Woodland Hills, CA 91364, USA; marilyn.geller@celiac.org; 3 Celiac Disease Center, Department of Medicine, Columbia University Medical Center, 180 Fort Washington Avenue, Suite 936, New York, NY 10032, USA; hmz2105@columbia.edu (H.M.Z.); pg11@cumc.columbia.edu (P.H.R.G.); 4 Deartment of Epidemiology, Mailman School of Public Health, Columbia University Medical Center, New York, NY 10032, USA

**Keywords:** Celiac Disease, depression, gluten-free diet, dietary adherence

## Abstract

Background: The prevalence of depression in celiac disease (CD) is high, and patients are often burdened socially and financially by a gluten-free diet. However, the relationship between depression, somatic symptoms and dietary adherence in CD is complex and poorly understood. We used a patient powered research network (iCureCeliac^®^) to explore the effect that depression has on patients’ symptomatic response to a gluten-free diet (GFD). Methods: We identified patients with biopsy-diagnosed celiac disease who answered questions pertaining to symptoms (Celiac Symptom Index (CSI)), GFD adherence (Celiac Dietary Adherence Test (CDAT)), and a 5-point, scaled question regarding depressive symptoms relating to patients’ celiac disease. We then measured the correlation between symptoms and adherence (CSI vs. CDAT) in patients with depression versus those without depression. We also tested for interaction of depression with regard to the association with symptoms using a multiple linear regression model. Results: Among 519 patients, 86% were female and the mean age was 40.9 years. 46% of patients indicated that they felt “somewhat,” “quite a bit,” or “very much” depressed because of their disorder. There was a moderate correlation between worsened celiac symptoms and poorer GFD adherence (*r* = 0.6, *p* < 0.0001). In those with a positive depression screen, there was a moderate correlation between worsening symptoms and worsening dietary adherence (*r* = 0.5, *p* < 0.0001) whereas in those without depression, the correlation was stronger (*r* = 0.64, *p* < 0.0001). We performed a linear regression analysis, which suggests that the relationship between CSI and CDAT is modified by depression. Conclusions: In patients with depressive symptoms related to their disorder, correlation between adherence and symptoms was weaker than those without depressive symptoms. This finding was confirmed with a linear regression analysis, showing that depressive symptoms may modify the effect of a GFD on celiac symptoms. Depressive symptoms may therefore mask the relationship between inadvertent gluten exposure and symptoms. Additional longitudinal and prospective studies are needed to further explore this potentially important finding.

## 1. Introduction

Celiac Disease (CD) is an autoimmune disorder that, by recent estimates, affects roughly 0.7% of the United States population [1]. The disease is caused by gut mucosal immune activation against gluten, a protein component of wheat, rye, and barley [2]. The clinical manifestations are numerous and often differ between adults and children. While potential new therapies are being studied, the only current treatment is strict avoidance of gluten in the diet [3]; a treatment that for many is expensive, socially isolating, and can result in anxiety about accidental ingestion [3].

There are many well-described neuropsychiatric manifestations of CD. Many studies have examined the association between CD and depression [4,5,6,7,8,9,10,11,12], anxiety [7,10], bipolar disorder [10], and schizophrenia [3,6]. The prevalence of depression in CD has been reported as anywhere between 6% and 57% [13], and a 2011 meta-analysis showed that depression is more common in CD than the general population [5]. However, other studies have demonstrated that the prevalence of depression in CD is the same as in the general population [14] and in other cohorts that suffer from chronic illness [5,15]. Many pathophysiologic mechanisms behind the CD-depression association have been postulated and studied including regional cerebral hypoperfusion [12], comorbid autoimmune thyroid disease [11], and decreased levels of cerebrospinal fluid monoamines in celiac patients, which improve with a gluten-free diet (GFD) [16,17]. The GFD can be expensive, burdensome, and socially isolating [3]. It is therefore conceivable that adhering to such a diet may be associated with worsening of affective symptoms. However, one cross-sectional study demonstrated an association between less severe depression scores and being on a gluten-free diet for more than five years [8] and another longitudinal study found improvements in quality of life after one year of treatment of a GFD, with less improvement in those who adhered poorly [18]. Simsek et al. (2015) identified improved depression scores in pediatric patients adherent to a GFD [19] as did Borghini et al. [20] in adult patients, where another study suggested improvement in anxiety only, with no change in depressive symptoms after one year of a GFD [21]. Furthermore, depressed patients who suffer from chronic illnesses have been found to be less compliant with treatment than non-depressed individuals [22]. A recent meta-analysis examined 8 cross-sectional studies and concluded that an association between poorer GFD adherence and self-reported depressive symptoms is likely [23]. Another study followed 66 patients, randomized to receive or not receive psychological support and found reduced depression scores and better adherence to their diet at six months in patients receiving psychological support [24]. This suggests that a relationship may exist between depressive symptoms and GFD adherence; however, the common use of self-reported depression and dietary adherence scales challenges the study of the topic. Furthermore, many of these studies do not specifically examine symptoms of CD. As many common CD symptoms may mimic those of depression, this remains a challenging relationship to evaluate scientifically.

In order to involve patients in clinical research, the Patient-Centered Outcomes Research Institute (PCORI) created the National Patient-Centered Clinical Research Network. As an outcome of this initiative, the Celiac Disease Foundation created iCureCeliac^®^, a patient-governed forum for clinical research. We implemented this tool to study the effect of self-reported depressive symptoms as relating to CD on the association between CD symptoms and adherence to a GFD. We hypothesized that the presence of depressive symptoms modifies the symptomatic response to a GFD.

## 2. Patients and Methods

We performed a cross-sectional analysis of pre-existing study data, utilizing the research questionnaire from iCureCeliac^®^ a patient-powered research network. Beginning in January 2016, the questionnaire was posted on the Celiac Disease Foundation website and reminders were periodically emailed to newsletter subscribers. Patients had the option to enter as much or as little data as they desired on an entirely voluntary basis with no financial incentive offered.

### 2.1. Inclusion Criteria

At the time of data analysis, the iCureCeliac^®^ questionnaire included data from 1724 individuals, with gluten-related disorders that included CD, non-celiac gluten sensitivity, dermatitis herpetiformis, wheat allergy, and self-diagnosed gluten-related disorder. We included patients of all ages who indicated in the questionnaire a diagnosis of CD, diagnosed at any age by endoscopy and biopsy and who answered the questions that applied to our study between the inception of the patient-powered research network on 30 January 2016 and 25 August 2016. A parent or legal guardian completed responses for children. Although per ACG and AGA guidelines [25,26], a diagnosis of CD is made using a combination of serology and a confirmatory biopsy of the small bowel to diagnose CD in patients with typical signs and symptoms, we assumed that biopsy-proven diagnoses were made in patients with symptoms and serology suggestive of celiac disease.

### 2.2. Data Collection

We collected basic demographic information including age, sex, education level, time to diagnosis from symptom onset, and degree of adherence to a gluten-free diet. We extracted data from one question asking patients about depression as it relates to their gluten-related disorder, phrased as follows: “I feel depressed because of my gluten-related disorder”. Five responses were offered ranging from “not at all” to “very much”. We considered the responses “somewhat”, “quite a bit”, and “very much” to indicate the presence of depressive symptoms, whereas “not at all” and “a little bit” to indicate the absence of depressive symptoms. A smaller subset of patients responded to the PROMIS (Patient Reported Outcomes Measurement Information System) Depression instrument. PROMIS is a set of validated, self-reported measures that evaluate various physical, psychological and social symptoms designed for use in adult and pediatric populations for research. The PROMIS Depression instruments include a complete 28-item assessment, as well as 4-, 6-, and 8-question short forms. Upon completion of the questionnaire, a raw score is generated, which corresponds to a T-score (based on a conversion table provided) with an instrument-specific range (41.0 to 79.4 for the 4-question short form used in the iCureCeliac^®^ questionnaire). The T score is compared to the population mean with standard errors provided.

We also extracted data from questions administered to questionnaire respondents relating to symptoms and dietary adherence, which comprised most of the Celiac Symptom Index (CSI) and Celiac Dietary Adherence Test (CDAT) questions outlined by Leffler et al. [27,28]. The CSI and CDAT are clinically oriented, easily administered, questionnaires with 16 and 7-items, respectively. The CSI performed well as a surrogate measure of disease activity [27] and the CDAT was shown to perform better than tissue transglutaminase titers for evaluating dietary adherence [28].

### 2.3. Data Analysis

The iCureCeliac^®^ questionnaire asked fifteen of the sixteen questions included in the CSI (missing question: “How much physical pain have you had during the last 4 weeks”?) and five out of the seven questions included in the CDAT (missing scaled questions: “I do not consider myself a failure” and “Before I do something, I carefully consider the consequences”). As not all of the CSI and CDAT questions were included in the initial iCureCeliac^®^ questionnaire, the instruments are incomplete. We therefore analyzed these responses out of a total of 75 and 25 possible points, respectively (compared with 80 and 35, respectively). Higher CSI scores correlate with more severe symptoms and higher CDAT scores correlate with poorer dietary adherence, as described by Leffler et al. [27,28].

We calculated correlation coefficients with 95% confidence intervals between: (1) adherence to a GFD (CDAT) and CD symptoms (CSI); (2) depression and CD symptoms (CSI); and (3) depression and adherence to a GFD (CDAT). We then stratified patients based on the presence of depressive symptoms so as to determine whether these symptoms modify the association between adherence to a GFD and CD symptoms. To formally test for interaction, we constructed a multiple linear regression model with CSI score as the outcome, and CDAT score, the presence of depression, and the interaction term (depression*CDAT) as the dependent variables.

As a means of validating our chosen screening question for depressive symptoms, we calculated a correlation coefficient to compare our question to the 4-item PROMIS depression score in the smaller subset of respondents who completed both sets of questions regarding depression. For all PROMIS Depression instruments, a T-score of 50 is equivalent to the mean of the general population with a standard deviation of 10. The 4-item depression short form has been validated against the Center for Epidemiologic Studies Depression Scale [29]. For the purpose of this study, patients with a raw score greater than or equal to 11 (corresponding to a T score of, ≥60.5 or 1 SD above the mean of the general population) were considered to have a positive depression screen.

We used the guidelines set forth by Evans [30] for interpretation of correlation coefficient values.

We used SAS version 9.4 (SAS Institute Inc. 2013, Cary, NC, USA) to calculate Pearson correlation coefficients for the variables listed above. Although the study was designed after data collection, our hypothesis was developed prior to data analysis. Informed consent was obtained from each patient prior to completion of the survey. This study conforms to the ethical guidelines set forth by the 1975 declaration of Helsinki and was approved by the Institutional Review Board of Columbia University Medical Center on 22 September 2016.

## 3. Results

We identified 519 patients with biopsy-diagnosed CD who met criteria for inclusion in our study. The characteristics of our study population are displayed in Table 1. The participants were predominantly female (86%). The mean age was 40.9 years (standard deviation (SD) ± 16.7), 26 patients were aged 16 years or less, 92% of our study population self-reported their race as white and 65% had completed at least one year of college-level education. Feeling depressed was reported by 46% of respondents (“very much” by 6.7%, “quite a bit” by 12.5%, and “somewhat” by 26.8%). The mean (±SD) CDAT score was 12.81 (±2.53, out of a 25-point scale, IQR = 16) and the mean (±SD) CSI score was 36.1 (±11.2 out of a 75-point scale, IQR = 11).

Depression had a weak correlation with worse symptoms (*r* = 0.35, 95% CI 0.26–0.43, *p* < 0.0001) and a weaker correlation with GFD adherence (*r* = 0.25, 95% CI 0.16–032, *p* < 0.0001). There was a moderate correlation between worse symptoms and poorer GFD adherence (*r* = 0.60, 95% CI 0.53–0.66, *p* < 0.0001).

**Figure 1:**
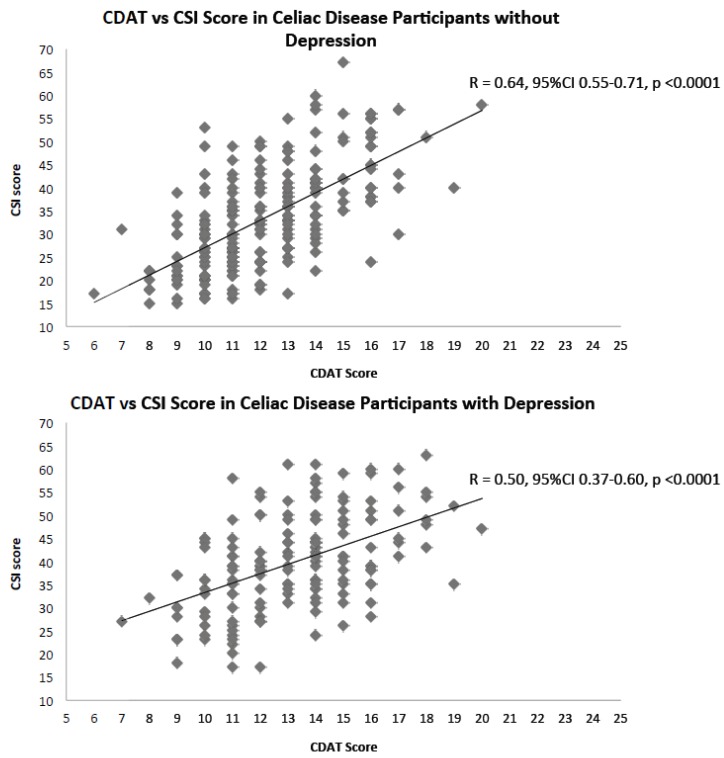
Celiac Dietary Adherence Test vs. Celiac Symptom Index Scores in patients with depressive symptoms versus those without depressive symptoms.

In patients with a positive depression screen, there was a moderate correlation between worsening symptoms and worsening dietary adherence (*r* = 0.50, 95% CI 0.37–0.60, *p* < 0.0001) whereas in those without depression, the correlation was still moderate, although stronger (*r* = 0.64, 95% CI 0.55–0.71, *p* < 0.0001). When patients <16 years of age were removed from the analysis (*n* = 370), the results were unchanged. The difference in CSI vs. CDAT scores in depressed vs. non-depressed patients is shown in Figure 1. When formally testing for interaction (*n* = 392), the beta coefficient for CDAT*depression was −0.935 (95% CI −1.65–−0.22, *p* = 0.0109), suggesting that the relationship between CSI and CDAT is modified by depression.

137 patients answered the 4-item PROMIS Depression instrument. Using a cutoff of one standard deviation above the mean, 24 patients (17.5%) met criteria for depression (T score ≥ 11) and 113 (82.5%) were not depressed (T score ≤ 10). The mean PROMIS raw score was 6.9 (SD ± 3.6) corresponding to a T score of 53.9 (SE 2.4). 91 patients (66.4%) scored below the mean and 46 (33.6%) patients scored above the mean. Our chosen depression screening question correlated moderately (*r* = 0.48, *p* < 0.0001) with the PROMIS score. Furthermore, in the same subset of patients, we found a similarly moderate correlation between symptoms and adherence in depressed patients (*r* = 0.46, *p* = 0.03) and moderate, although stronger correlation in patients who were not depressed (*r* = 0.57, *p* < 0.0001).

## 4. Discussion

In this cross-sectional study of 519 patients with biopsy-diagnosed CD, we found that self-reported depressive symptoms were common, occurring in 46% of the respondents. We also found that the relationship between CD symptoms and adherence to a GFD may be attenuated based on the presence of depressive symptoms related to having celiac disease. In those without depressive symptoms, the correlation between symptoms and dietary adherence was moderate, although stronger than in those with a positive depression screen. This was further supported by our regression analysis demonstrating significant interaction of depression on the relationship between GFD adherence and CD symptoms. These results suggest that the presence of depressive symptoms may attenuate the relationship between adherence to a GFD and symptoms of CD as those with depression may have less symptomatic relief from better adherence to a GFD than those without depressive symptoms.

We also found a weak correlation between higher levels of depression (based on our screening question) and poor dietary adherence. The association between adherence to a GFD and depression was further evaluated in a recent meta-analysis, which found a likely association between poor GFD adherence and worsened depressive symptoms. Furthermore, Addolorato et al. demonstrated that CD patients who received six months of psychological counseling had better adherence to their diets and lower rates of depression [24]. The weak correlation found in our study does not support the relationship between poor dietary adherence and depression described by others [23]. Rather, our results suggest that depression affects the relationship between adherence to a GFD and CD symptoms. One possible, reason for this effect modification is that patients with depression may have their symptoms driven or exacerbated by factors other than gluten exposure, which may manifest physically. However, there are many other potential causes of symptoms in CD including functional bowel disorders, inadvertent gluten exposure, and concomitant food intolerances. The relationship between celiac symptoms and depressive symptoms is complex and identifying which symptoms are attributable to each process can be challenging for clinicians. 

This study has a number of limitations, some of which are inherent to its retrospective, self-reported and observational nature. The study was compiled from a voluntary questionnaire in a patient-powered research network. Although there was no financial incentive to complete the questionnaire, the study population represents a self-selected cohort of patients, who may be more aware of their illnesses and therefore may be more adherent to a GFD than the general CD population. This is supported by the high percentage (87%) of patients reporting they “always” adhere to a GFD. Furthermore, the majority of our research cohort was comprised of white, highly educated females. Unfortunately, the initial questionnaire did not contain all of the questions that make up the CSI and the CDAT, so each of these instruments is incomplete. However, for the purpose of our study, the trend (higher CDAT scores representing worse dietary adherence and higher CSI scores representing more symptomatic patients) was sufficient to evaluate our research question. Moreover, we feel that the correlation of higher CD symptom scores (worse symptoms) with poorer dietary adherence suggests validity, despite the abbreviated indices available.

Another limitation was in our utilization of a single question to screen patients for depression. Our screening question demonstrated moderate correlation with the validated 4-item PROMIS Depression instrument. However the PROMIS questions were completed by only a small subset of participants, which may lead to selection for more depressed patients. By PROMIS criteria, the prevalence of depression in our study was 17.5%. In contrast, using our 1-item screening question, depressive symptoms in our study were more common, with nearly half of respondents (46%) reporting that they felt at least “somewhat” depressed with regards to their disease. This difference may be due to the use of the chosen depression screening question which may signify the emotional reaction to having CD as opposed to clinical depression. Addolorato et al. [21] demonstrated a 57% prevalence of depression in their study, but others have shown the prevalence of active depressive symptoms in CD patients to be lower [4]. The prevalence of depression found in our study should therefore be interpreted with caution, as it may represent a self-selecting, more symptomatic population. Furthermore, these results represent self-reported data at a point in time, and not diagnoses made by health care professionals. 

## 5. Conclusions

In conclusion, our analysis of data provided through a CD patient-powered research network, suggests that depression may modify the relationship between adherence to a GFD and the presence of symptoms in CD. In patients who screened positive for depressive symptoms, the correlation between CD symptoms and response to a GFD was attenuated. These findings support that screening for depression should be considered in patients with CD, as those with depression may have less improvement in symptoms despite better adherence to the diet than those without depression. The study of this topic remains a challenge due to a lack of longitudinal and prospective studies. The relationship between dietary adherence and depression remains complex and the direction of the association remains unclear [23]. As suggested previously, patients receiving psychological counseling may show better adherence to a GFD [24], but further prospective and randomized studies are needed to explore whether or not treatment for depression improves how patients respond symptomatically to a GFD. Ideally, future studies will encompass more diverse groups that represent the true CD population in the United States with validated measures of depression to further substantiate these findings. Our study also provides demonstrable evidence of the value of patient-powered research networks in the ongoing study of celiac disease.

## Figures and Tables

**Table 1:** Patient characteristics of our study population. As the questionnaire was voluntary, some questions are missing responses from all 519 participants.

Characteristic		Study Population (*n* = 519)
**Gender**		
Male		74 (14%)
Female		445 (86%)
**Age (mean ± SD)**		40.9 (±16.7)
**Race/Ethnicity (*n* = 509)**		
Black/African American		3 (0.6%)
Latino/Hispanic		8 (1.6%)
White		469 (92.1%)
Other (including more than 1)		29 (5.7%)
**Highest education level (*n* = 207)**		
Less than a college degree		60 (29%)
College degree or equivalent		85 (41.1%)
Master’s degree or degree beyond Bachelor’s degree		49 (23.7%)
Doctorate degree		13 (6.3%)
**Currently working (*n* = 209)**		
Yes		149 (71.3%)
No—on disability		5 (2.4%)
No—retired		24 (11.5%)
No—other		24 (11.5%)
**Degree of Strict Gluten Free Diet Adherence (*n* = 516)**		
Always		453 (87.3%)
Often		51 (9.8%)
Sometimes		9 (1.7%)
Rarely		2 (0.4%)
Never		1 (0.2%)
Skipped/Missing		3 (0.6%)
**Length of time from symptom onset to Celiac Disease diagnosis**		
<5 years		356 (68.6%)
5–15 years		67 (12.9%)
>15 years		38 (7.3%)
Don’t Know/missing		58 (11.2%)
**Depressed (*n* = 519)**		
Yes	Very Much	35 (6.7%)
	Quite A bit	65 (12.5%)
	Somewhat	139 (26.8%)
No	A little bit	157 (30.3%)
	Not at all	125 (23.7%)
**Celiac Dietary Adherence Test (*n* = 519)**		
Mean (±SD)		12.8 (±2.5)
First Quartile		3
Median		13
Third Quartile		14
Interquartile Range		11
**Celiac Symptom Index (*n* = 392)**		
Mean (±SD)		36.1 (±11.2)
First Quartile		27
Median		36
Third Quartile		43
Interquartile Range		16
